# Robot-assisted direct repair of spondylolysis: A case report

**DOI:** 10.1097/MD.0000000000018944

**Published:** 2020-01-24

**Authors:** Wei Tian, Qi Zhang, Xiao-Guang Han, Qiang Yuan, Da He, Ya-Jun Liu

**Affiliations:** a Department of Spine Surgery, Beijing Jishuitan Hospital; b Department of Spine Surgery, Peking University Fourth School of Clinical Medicine, Beijing, China.

**Keywords:** direct repair, intralaminar screw fixation, robot-assisted surgery, robotic system, spondylolysis

## Abstract

**Introduction:**

Direct repair of the pars defect in lumbar spondylolysis is an effective surgical procedure, but it is technically challenging. We assessed the feasibility of a new robotic system for intralaminar screw fixation of spondylolysis.

**Patient concerns:**

A 26-year-old man complained about frequent low back pain after failed conservative treatments.

**Diagnosis:**

The lumbar computed tomography images demonstrated the presence of bilateral spondylolysis at the L5 level, with no spondylolisthesis.

**Interventions:**

We performed one surgery of direct intralaminar screw fixation under the guidance of the TiRobot system. The trajectory of the screw was planned based on intraoperative 3-dimensional radiographic images. Then, the robotic arm spontaneously moved to guide the guide wires and screw insertion.

**Outcomes:**

Bilateral L5 intralaminar screws were safely and accurately placed. No intraoperative complications occurred. Postoperative computed tomography showed good radiological results, without cortical perforation.

**Conclusion:**

We report the first case of robot-assisted direct intralaminar screw fixation for spondylolysis using the TiRobot system. Robotic guidance for direct repair of spondylolysis could be feasible.

## 1 Introduction

Spondylolysis occurs in nearly 6% of the general population.^[1]^ This pathology causes low back pain in adolescents or young adults and is often associated with spondylolisthesis. Direct repair of the pars defect has been proved to be an effective surgical procedure in lumbar spondylolysis.^[2–4]^ This motion-preserving technique has also demonstrated better biomechanical results.^[5]^ In recent years, we applied a new robot system (TiRobot, TINAVI Medical Technologies, Beijing, China) in clinical orthopedic surgery. The accuracy of this novel system in pedicle screw insertion was above 95%.^[6]^ We present the first case of robot-assisted (RA) direct intralaminar screw fixation for spondylolysis performed under the guidance of the TiRobot system.

## 2 Case presentation

A 26-year-old male complained of frequent low back pain for 10 months without any significant radicular pain. He had failed conservative treatments after 8 months. On neurological examination, there was no sensory-motor deficit.

**Figure 1:**
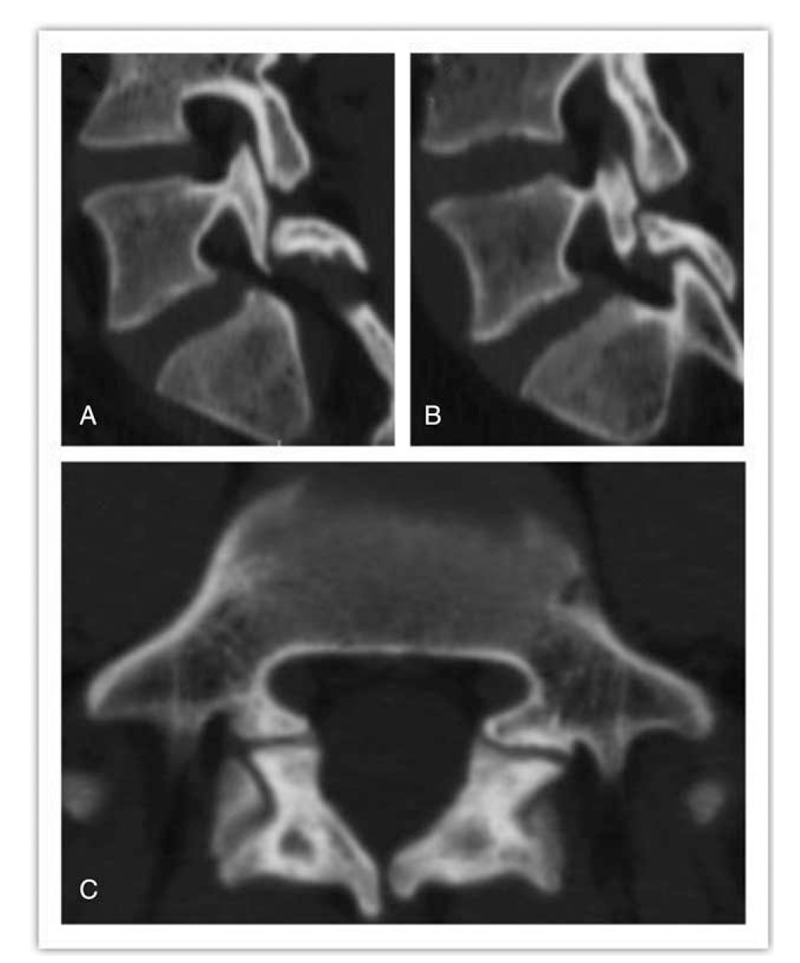
Axial and sagittal computed tomography images showing the presence of bilateral spondylolysis at the L5 level.

The lumbar computed tomography images demonstrated the presence of bilateral spondylolysis at the L5 level (Fig. 1), with no spondylolisthesis. Magnetic resonance imaging showed no lumbar disc degeneration or herniation.

**Figure 2:**
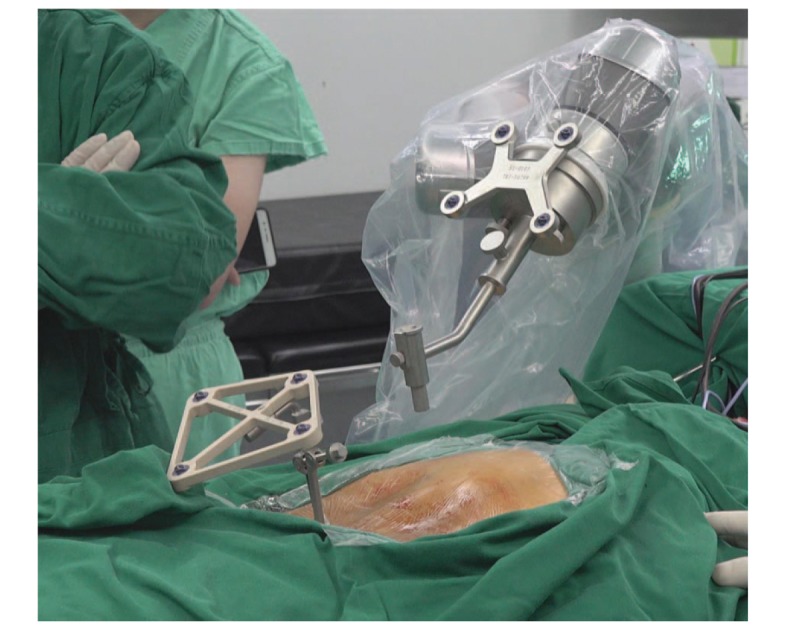
The surgical field in robot-assisted surgery.

The patient has provided informed consent for publication of the case. After approval by the ethics committee and informed consent, a surgery of RA direct intralaminar screw fixation was scheduled. The patient was placed in a prone position after general anesthesia. A standard posterior midline exposure of the involved vertebra was made. The fibrous tissue and the sclerotic bone in the pars defect were cleaned till bleeding bony surface. After the reference tracker was mounted at the spinous process of L4, a set of 3-dimensional images was acquired by an intraoperative C-arm scan. Based on these images, intraoperative planning of the intralaminar screws was performed using the TiRobot system. The robot could spontaneously move to the chosen trajectory. Then, we inserted the guide wires through the cannula on the robotic arm under real-time navigation monitoring and adjustment (Fig. 2). Two 4.5-mm cannulated screws were inserted along the guided path across the defect. A re-scan was performed to confirm the position of the screws. This surgery took a total of 2 h, with a blood loss of 10 mL.

**Figure 3:**
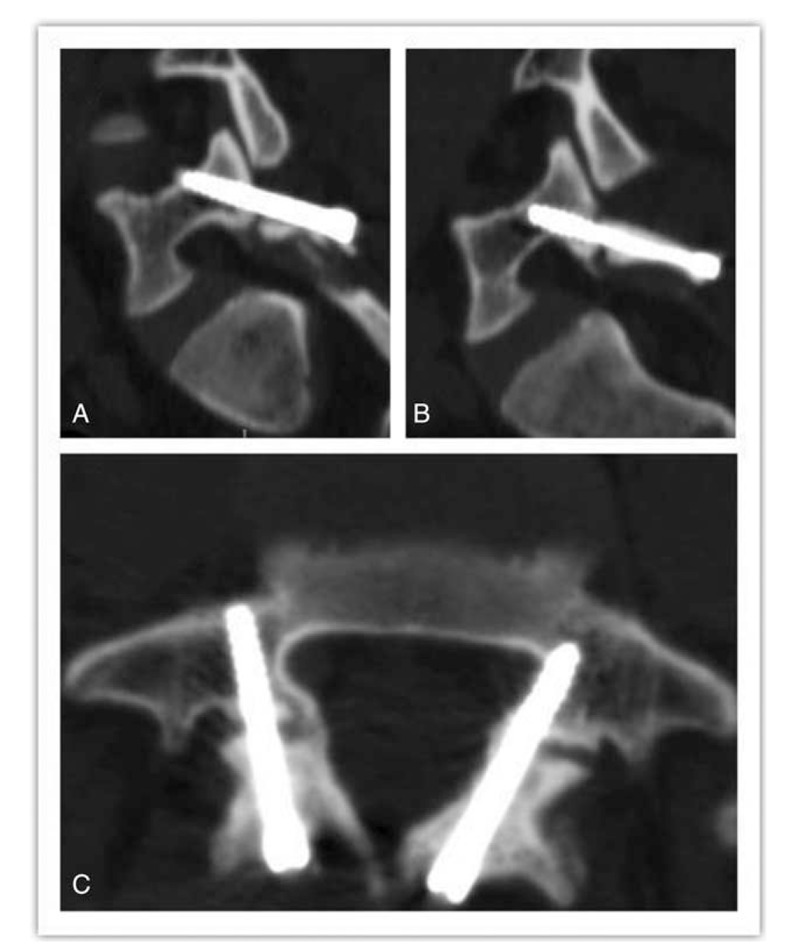
Axial and sagittal postoperative computed tomography images of the patient.

Immediate postoperative recovery was achieved, with no neurological deficit and other complications. A soft brace patient was worn after ambulation, and no sports were allowed for 3 months. A postoperative computed tomography scan showed acceptable screw positions (Fig. 3). The pain status measured by the visual analogue score was 1 at postoperative 6 months. The Oswestry Disability Index was 6 at postoperative 6 months.

## 3 Discussion

Orthopedic surgeons have limitations in vision and manipulation, which restricted the development of minimally invasive spine surgery. Unable to see structures beneath and insufficient force control could increase the risk of iatrogenic injury during spine surgery.^[7]^ Therefore, researchers had endeavored to develop the surgical robot systems for spine surgery. Some robot systems have been successfully used in clinical surgeries to insert the pedicle screws,^[8–11]^ but other applications of the robot in spine surgery has been reported infrequently. Hence, we presented the first case of RA direct intralaminar screw fixation for spondylolysis.

Several techniques have been applied for the surgical treatment of a pars defect. Direct repair of the defect in spondylolisthesis, by which screws are placed directly through the fractured pars, was first described by Buck in 1970.^[2]^ This procedure aims to obtain the bone union of the isthmus, to restore the stability of the spine and to prevent the development of spondylolisthesis. This technique was indicated for patients aged <30 years, with or without grade 1 spondylolisthesis, with a normal disc and facet joint confirmed by magnetic resonance imaging.^[12]^ Debnath et al^[13]^ reported that 95% returned to sports after this procedure. Menga et al^[3]^ described that 76% (19/25 athletes) returned to competitive sports with this technique. However, on the other hand, it is a technically demanding procedure.^[4,14]^ In response, we applied this new surgical robot to reduce the operating difficulty, thus verify the feasibility of RA direct repair of pars defect.

This new surgical robot was reported that could provide accurate positioning, adequate steadiness, and repeatability in surgery.^[15–17]^ Higher accuracy and minimal invasion were the advantages of RA technology when compared to traditional methods. Traditional methods require extensive exposure of paravertebral muscles and ligaments, to find the correct orientation. However, using the RA technique, the direction of screws is not tricky through a percutaneous approach. Decreased soft tissue dissection has been shown to decrease infection, muscle atrophy, length of stay, and blood loss.^[18–20]^ Besides, the RA technique could decrease the operating duration by rapid planning and placement of the screws.

This study has some limitations. First, only one case of intralaminar screw fixation was completed. A much larger number of patients and a control group would be needed to assess the safety and accuracy of this RA technique. Second, long-term follow-up with patient-reported outcomes would be required to evaluate the postoperative outcomes and bone union.

We report the first case of RA direct intralaminar screw fixation for spondylolysis using the TiRobot system based on intraoperative 3-dimensional images. The use of robotic guidance in the direct repair of spondylolysis could be feasible.

## Author contributions

**Conceptualization:** Wei Tian, Qi Zhang, Ya-Jun Liu.

**Data curation:** Xiao-Guang Han, Qiang Yuan.

**Formal analysis:** Qi Zhang, Xiao-Guang Han, Da He.

**Funding acquisition:** Wei Tian, Ya-Jun Liu.

**Methodology:** Qi Zhang, Xiao-Guang Han, Qiang Yuan.

**Project administration:** Wei Tian, Da He, Ya-Jun Liu.

**Resources:** Qiang Yuan.

**Software:** Qi Zhang.

**Supervision:** Wei Tian.

**Validation:** Xiao-Guang Han, Qiang Yuan.

**Visualization:** Ya-Jun Liu.

**Writing – original draft:** Qi Zhang.

**Writing – review & editing:** Wei Tian, Qiang Yuan, Da He, Ya-Jun Liu.
